# Albuminuria and Premature Atherosclerotic Cardiovascular Disease in U.S. Adults: A Cross-Sectional Analysis of the National Health and Nutrition Examination Survey, 2011-2018

**DOI:** 10.7759/cureus.108287

**Published:** 2026-05-05

**Authors:** Helen Oletu, Akinyele Oladimeji, Gift Ojukwu, Ifunanya R Ekeocha, Steve Okwu, Azeberoje Osueni, Emeka K Okobi, Francis Okobi, Onyekachi O Anya, Nnaemeka H Ugwuchukwu, Olaoluwa M Adeyemi

**Affiliations:** 1 Internal Medicine, North Knoxville Medical Center, Powell, USA; 2 Family Medicine, Alberta Health Services, Edmonton, CAN; 3 General Practice, Leeds Teaching Hospital, Leeds, GBR; 4 Internal Medicine, Nnamdi Azikiwe University Teaching Hospital, Nnewi, NGA; 5 Cardiothoracic Surgery, Beacon Hospital, Dublin, IRL; 6 Gastroenterology and Hepatology, Lagos University Teaching Hospital, Lagos, NGA; 7 Maxillofacial Surgery, Ahmadu Bello University Teaching Hospital Zaria, Kaduna, NGA; 8 Research and Development, Covance, Hanover, USA; 9 Internal Medicine, Legacy Salmon Creek Medical Center, Vancouver, USA; 10 Oncology, Antrim Area Hospital, Antrim, GBR; 11 Internal Medicine, Advocate Christ Medical Center, Chicago, USA

**Keywords:** albuminuria, cardiovascular risk, chronic kidney disease, epidemiology, nhanes, premature atherosclerotic cardiovascular disease

## Abstract

Background: Albuminuria is recognized as a marker of vascular and renal dysfunction and has been linked to adverse cardiovascular outcomes. Its relationship with premature atherosclerotic cardiovascular disease (ASCVD) in the general population remains less clearly defined.

Objective: This study examined the association between albuminuria and premature ASCVD among U.S. adults using nationally representative data from the National Health and Nutrition Examination Survey (NHANES) 2011-2018.

Methods: A cross-sectional analysis was conducted using data from the NHANES 2011 to 2018. The study included adults aged 18 years and older with complete data on exposure, outcome, and covariates. Albuminuria was defined using the urinary albumin-to-creatinine ratio (ACR). Premature ASCVD was defined based on self-reported myocardial infarction, stroke, or coronary heart disease occurring at younger ages. A survey-weighted logistic regression model was used to assess the association while accounting for the complex sampling design.

Results: Albuminuria was associated with higher odds of premature ASCVD after adjustment for demographic and clinical factors. Participants with albuminuria also had a higher burden of hypertension, diabetes, smoking, and chronic kidney disease (CKD). Increasing age and lower income were associated with higher odds of premature disease, while smoking showed a strong association.

Conclusion: Albuminuria is associated with premature ASCVD and may serve as a useful marker for early cardiovascular risk identification. These findings support its role in clinical risk assessment and highlight the need for further longitudinal research.

## Introduction

Cardiovascular disease (CVD) is the number one cause of morbidity and mortality in the United States, as it is a trend and an increasing problem leading to untimely deaths and medical expenses [1]. Recent developments show that there is a worrying tendency to develop atherosclerotic CVD (ASCVD) in younger people (men younger than 55 years and women younger than 65 years) [2]. The focus on sex- and age-specific strategies on ASCVD has led to the emergence of programs, campaigns, and guidelines on this problem [3]. The disparities in treatment and management of CVD and the risk factors have traditionally been discussed as racial and ethnic, as one of the key contributors to health inequity [4].

The microvascular and macrovascular endothelial dysfunction is believed to be a biomarker of albuminuria [5, 6]. Although albuminuria is already identified as a biomarker of generalized endothelial dysfunction, information regarding the assessment of albuminuria in chronic obstructive pulmonary disease (COPD) patients and its correlation with disease outcome measures is lacking [6]. ASCVD also predisposes to all-cause mortality and CVD and deterioration to renal failure, despite the presence of conventional risk factors, including hypertension and diabetes mellitus [7]. Albuminuria is a strong forecast of further events, and it is possible to say that one should draw attention to the data on albuminuria, along with kidney activity, in this high-risk population [8]. Albuminuria screening is most commonly used in clinical practice when it comes to staging kidney disease or screening individuals with hypertension or diabetes due to end-stage organ damage [9]. Adults who had high albuminuria in the low range (urine albumin-to-creatinine ratio (ACR) <30 mg/g) and none of the significant cardiovascular risk factors had increased risks of cardiovascular and all-cause mortality. The risk was on a linear relation to the increasing level of albuminuria [10, 11].

Endothelial dysfunction, which is an intermediate cardiovascular outcome [4], is an important factor in atherosclerosis and vascular lesion formation [12]. Oxidative stress and inflammation have been said to be key contributors in the pathogenesis of diabetic complications such as renal and CVD [13]. Diastolic dysfunction was observed to be linked to low-grade albuminuria in hypertensive patients, more so when the patient is below 70 years of age and in those with less than 15 years of hypertension [14, 15].

Although there is increasing evidence that albuminuria is a potential predictor of cardiovascular outcomes, little has been done to specifically determine its relationship with premature ASCVD in the general U.S. population [16]. Numerous past research studies have focused on older people or those who already have known comorbidities, which may fail to represent the problem of albuminuria in younger adults or during the earlier phases of the disease [17]. Although diabetes and hypertension appear to be significant factors contributing to increased prevalence of albuminuria across all racial/ethnic groups, additional independent risk in the context of these known risk factors appears to exist for American Indian/Alaska Native patients [18].

The study utilizes the National Health and Nutrition Examination Survey (NHANES). Utilizing the NHANES dataset makes it possible to evaluate the relationship between albuminuria and premature ASCVD in the US, accounting for a wide range of population groups [19]. Using this data, one can test the relationship between albuminuria and premature ASCVD with a large number of possible confounding factors, such as age, sex, race, comorbidities, and socioeconomic factors [20-21].

This study aimed to examine the association between albuminuria and premature ASCVD among U.S. adults using nationally representative data from NHANES 2011-2018. In conclusion, the findings of the study will add to the existing literature on the role of albuminuria and premature ASCVD.

## Materials and methods

Study design and data source

The current analysis used a cross-sectional methodology that utilized data from NHANES collected from 2011 to 2018 [21]. NHANES represents a nationally representative survey of the US civilian population residing in non-institutional settings and employs a complex multistage probability-based sample design to obtain the sample. Survey data are collected from respondents through a household interview process and through a mobile examination center using standardized forms of physical examination and laboratory testing [21]. To enhance statistical power and representativeness of the resulting dataset, four NHANES survey cycles were combined.

Study population

The study population included adults aged 18 years and older with complete data on albuminuria, premature ASCVD, and selected covariates. Participants with missing data on key variables, including albuminuria, body mass index (BMI), hypertension status, diabetes status, smoking status, kidney function, or income-to-poverty ratio, were excluded to ensure a consistent analytic sample. After applying these criteria, a final sample of 19,577 participants was included in the analysis, representing an estimated 213,050,904 U.S. adults after application of survey weights.

Variables and measures

The primary variable measured was albuminuria, which is defined as the presence of a urinary ACR in a single spot urine specimen. An ACR value of 30 mg/g or above was used to identify the presence of albuminuria, with values lower than this used to identify the absence of albuminuria. The primary endpoint of this study was the presence of premature ASCVD, as determined by a self-reported history of myocardial infarction, stroke, or coronary heart disease, as indicated by answers to questions MCQ160E, MCQ160F, and MCQ160C from the questionnaire [21]. The age of diagnosis for each condition was determined using variables MCQ180E, MCQ180F, and MCQ180C. Premature ASCVD was defined as the occurrence of any of the above conditions prior to age 55 in males and age 65 in females. Other covariates included demographic and clinical data, with age treated as a continuous variable. The sex of participants was categorized based on the NHANES variable RIAGENDR (male or female). Race/ethnicity categories were defined according to the NHANES classification and were used as provided in the original dataset. The classifications were as follows: Mexican American, Other Hispanic, Non-Hispanic White, Non-Hispanic Black, Non-Hispanic Asian, and Other Race, based on the RIDRETH3 variable, with Non-Hispanic White as the reference group in regression models. Socioeconomic status was based on the family income to poverty ratio, calculated from the INDFMPIR variable. Blood pressure measurements were used to calculate an average systolic and diastolic blood pressure from BPXSY1 - BPXSY3 and BPXDI1 - BPXDI3, respectively. Diabetes was defined as being told by a physician or other health professional that one has diabetes, which was reported on DIQ010. The smoking status was defined as having smoked 100 or more cigarettes in one's lifetime. This was collected using SMQ020, asking about whether one smoked 100 or more cigarettes in one's lifetime. We assessed kidney function with an estimated glomerular filtration rate (eGFR) based on serum creatinine (LBXSCR), age, and sex. Chronic kidney disease (CKD) was defined as an eGFR less than 60 mL/min/1.73 m².

Missing data

A complete case analysis approach was used due to the cross-sectional nature of NHANES data and to maintain consistency with established analytic practices; the potential for bias due to missing data is acknowledged. Sensitivity analyses were not performed, as the exposure and outcome variables were defined using standardized NHANES criteria with limited alternative validated categorizations. Participants with missing values in any of the variables required for the main models were excluded to ensure a consistent analytic sample. Missingness was low for most variables, including albuminuria at 4.29%, body mass index at 5.71%, hypertension at 4.29%, and smoking status at 0.77%. The income-to-poverty ratio had the highest proportion of missing data at 10.53%. No missing data were observed for the outcome variable or key demographic variables. This approach avoided variation in sample size across models and ensured consistency in the final analytic cohort.

Statistical analysis

The analysis of the data was consistent with the NHANES complex survey design by constructing sampling weights for all analyses, accounting for primary sampling units and strata. Weighted descriptive statistics were computed to describe the sample population based on albuminuria status. For continuous variables, means and standard deviations were reported, while categorical variables were reported as weighted proportions. Comparisons of the two groups based on the dependent outcome variable were made using survey-adjusted t-tests for continuous variables and survey-adjusted chi-square tests for categorical variables. A multivariate weighted logistic regression model was developed to assess the likelihood of developing premature ASCVD based on an individual's albuminuria status after controlling for demographic, clinical, and behavioral factors, including BMI, hypertension, diabetes, tobacco use, income-to-poverty ratio, and CKD. Multicollinearity among independent variables was assessed using variance inflation factors (VIF), with values ranging from 1.06 to 2.56 (mean VIF 1.44), indicating no significant multicollinearity. Model assumptions were evaluated prior to analysis. A two-tailed p-value of < 0.05 was used for statistical significance. Data cleaning, preparation, and analysis were done using Stata version 18 (StataCorp LLC, College Station, TX) [22].

Ethical considerations

NHANES protocols are approved by the National Center for Health Statistics Research Ethics Review Board, and all participants provide written informed consent. The present study used publicly available deidentified data and did not require additional ethical approval.

## Results

Table 1 below presents the weighted baseline characteristics of U.S. adults stratified by their albuminuria status.

**Table 1:** Weighted baseline characteristics of U.S. adults by albuminuria status, NHANES 2011 to 2018(n=19,577;N=213,050,904) Values are presented as weighted mean and standard deviation (SD) for continuous variables and weighted counts with column percentages for categorical variables. Percentages are column percentages. Survey-adjusted t tests were used for continuous variables, and survey-adjusted chi-square tests were used for categorical variables. ASCVD: atherosclerotic cardiovascular disease; CKD: chronic kidney disease; HTN: hypertension; NHANES: National Health and Nutrition Examination Survey; n: sample size; N: weighted population size; (–): intentionally left blank. The asterisk(*) indicates significance at 0.05. This table was generated and compiled by the authors using Stata version 18 (StataCorp LLC, College Station, TX, USA) [22]. Race/ethnicity categories are defined according to the NHANES classification and have been used as provided in the original dataset.

Characteristic	No albuminuria (N = 189,496,127)	Albuminuria (N = 23,554,777)	Test statistic	p-value
Age (years), mean ± SD	46.26 ± 16.44	54.99 ± 20.84	t = -17.65	<0.001*
Body mass index (kg/m²), mean ± SD	29.12 ± 6.75	30.57 ± 9.18	t = -6.74	<0.001*
Income-to-poverty ratio, mean ± SD	3.02 ± 1.63	2.51 ± 1.83	t = 9.21	<0.001*
Gender, n (%)	–	–	F = 7.43	0.008*
Male	92,215,486 (48.6%)	10,436,186 (44.3%)	–	–
Female	97,280,641 (51.4%)	13,118,591 (55.7%)	–	–
Race/Ethnicity, n (%)	–	–	F = 10.33	<0.001*
Mexican American	15,578,311 (8.2%)	2,081,820 (8.8%)	–	–
Other Hispanic	11,606,593 (6.1%)	1,353,208 (5.7%)	–	–
Non-Hispanic White	125,768,162 (66.4%)	14,319,842 (60.8%)	–	–
Non-Hispanic Black	19,987,904 (10.5%)	3,603,566 (15.3%)	–	–
Non-Hispanic Asian	10,126,387 (5.3%)	1,267,219 (5.4%)	–	–
Other Race	6,428,770 (3.4%)	929,122 (3.9%)	–	–
Hypertension, n (%)	–	–	F = 203.16	<0.001*
No HTN	100,438,935 (53.0%)	7,086,103 (30.1%)	–	–
HTN	89,057,192 (47.0%)	16,468,674 (69.9%)	–	–
Diabetes, n (%)	–	–	F = 599.98	<0.001*
No Diabetes	174,332,178 (92.0%)	16,929,807 (71.9%)	–	–
Diabetes	15,163,949 (8.0%)	6,624,970 (28.1%)	–	–
Smoking status, n (%)	–	–	F = 39.66	<0.001*
Never smoker	109,299,402 (57.7%)	11,799,908 (50.1%)	–	–
Ever smoker	80,196,725 (42.3%)	11,754,869 (49.9%)	–	–
CKD, n (%)	–	–	F = 428.52	<0.001*
No CKD	181,743,866 (95.9%)	19,495,971 (82.8%)	–	–
CKD	7,752,261 (4.1%)	4,058,807 (17.2%)	–	–
Premature ASCVD, n (%)	–	–	F = 137.25	<0.001*
No ASCVD	185,219,387 (97.7%)	22,076,917 (93.7%)	–	–
Premature ASCVD	4,276,740 (2.3%)	1,477,860 (6.3%)	–	–

Based on the findings above, it's evident that there are clear differences between participants with and without albuminuria. Individuals with albuminuria were older, with a mean age of 54.99 (20.84) years compared to 46.26 (16.44) years among those without albuminuria, and this difference was statistically significant (p < 0.001). BMI was higher in the albuminuria group, with a mean of 30.57 (9.18) kg/m² compared to 29.12 (6.75) kg/m², and the income-to-poverty ratio was lower, indicating lower socioeconomic status. A higher proportion of females was observed in the albuminuria group (13,118,591, or 56%), while males were more frequent in the non-albuminuria group (92,215,486, or 49%). Race and ethnicity distributions differed significantly, with a higher proportion of non-Hispanic Black participants among those with albuminuria and a lower proportion of non-Hispanic White participants. Clinical conditions were more common in participants with albuminuria. Hypertension was present in 16,468,674 (70%) participants of the albuminuria group, compared to 89,057,192 (47%) in those without albuminuria. Diabetes was also more frequent, affecting 6,624,970 (28%) participants in the albuminuria group compared to 15,163,949 (8%) in the non-albuminuria group. Ever smoking was reported by 11,754,869 (50%) of participants with albuminuria compared to 80,196,725 (42%) without albuminuria. CKD was markedly more prevalent in the albuminuria group, with 4,058,807 (17%) participants compared to 7,752,261 (4%) in those without albuminuria. Premature ASCVD was more common among participants with albuminuria, with a prevalence of 1,477,860 (6%) compared to 4,276,740 (2%) in those without albuminuria. All these differences were statistically significant (p < 0.001).

Table 2 presents the survey-weighted logistic regression analysis examining the association between albuminuria and premature ASCVD.

**Table 2:** Survey-weighted logistic regression analysis of factors associated with premature ASCVD among U.S. adults, NHANES 2011 to 2018 Results are presented as odds ratios with 95% confidence intervals. All models account for the NHANES complex survey design using sampling weights, strata, and primary sampling units. The reference group for race and ethnicity is non-Hispanic White, and for smoking is never smoker. ASCVD: atherosclerotic cardiovascular disease; NHANES: National Health and Nutrition Examination Survey; (–): intentionally left blank. The asterisk (*) indicates significance at 0.05. This table was generated and compiled by the authors using Stata version 18 (StataCorp LLC, College Station, TX, USA) [22]. Race/ethnicity categories are defined according to the NHANES classification and have been used as provided in the original dataset.

Characteristic	Adjusted odds ratio (95% CI)	p-value
Albuminuria	–	–
Yes vs No	1.41 (1.15 – 1.74)	0.001*
Age (per year)	1.03 (1.02 – 1.04)	<0.001*
Income-to-poverty ratio	0.80 (0.74 – 0.88)	<0.001*
Body Mass Index (per kg/m²)	1.02 (1.00 – 1.03)	0.035*
Gender	–	–
Female vs Male	1.21 (0.92 – 1.60)	0.160
Race/Ethnicity	–	–
Mexican American vs Non-Hispanic White	0.77 (0.56 – 1.06)	0.103
Other Hispanic vs Non-Hispanic White	0.98 (0.72 – 1.34)	0.899
Non-Hispanic Black vs Non-Hispanic White	1.16 (0.92 – 1.46)	0.203
Non-Hispanic Asian vs Non-Hispanic White	0.78 (0.52 – 1.16)	0.216
Other Race vs Non-Hispanic White	1.90 (1.12 – 3.21)	0.018*
Hypertension	–	–
Yes vs No	1.79 (1.34 – 2.40)	<0.001*
Diabetes		
Yes vs No	1.75 (1.32 – 2.32)	<0.001*
Smoking	–	–
Ever vs Never	2.18 (1.73 – 2.75)	<0.001*
Chronic Kidney Disease	–	–
Yes vs No	1.38 (0.99 – 1.92)	0.058

The results indicate that albuminuria was a strong predictor of increased risk of developing premature ASCVD (adjusted OR (aOR) =1.41, 95% CI: 1.15-1.74, p=0.001). In addition, age was also significantly associated with increased odds of developing premature ASCVD; for every year older a participant was, there was a 3% increased likelihood (aOR=1.03, 95% CI:1.02-1.04, p<0.001). An increase in the income to poverty ratio of the participant also decreased the odds of developing ASCVD early in life (aOR=0.80,95% CI:0.74-0.88, p<0.001). BMI had a small but significant positive relationship with ASCVD risk. Female sex did not significantly affect the risk of developing ASCVD before age 65 (aOR=1.21, 95% CI:0.92-1.60, p=0.160). There was not a significant difference between the majority of race/ethnicity categories compared to non-Hispanic White participants for ASCVD risk, except for participants identified as ‘other race’ who had significantly higher risk of ASCVD before age 65 (aOR=1.90, 95% CI:1.12-3.21, p=0.018). Hypertension, diabetes, and smoking had a very strong relationship with increasing the odds of developing ASCVD before age 65. CKD was positively associated with premature ASCVD, but did not meet the level of statistical significance.

**Figure 1:**
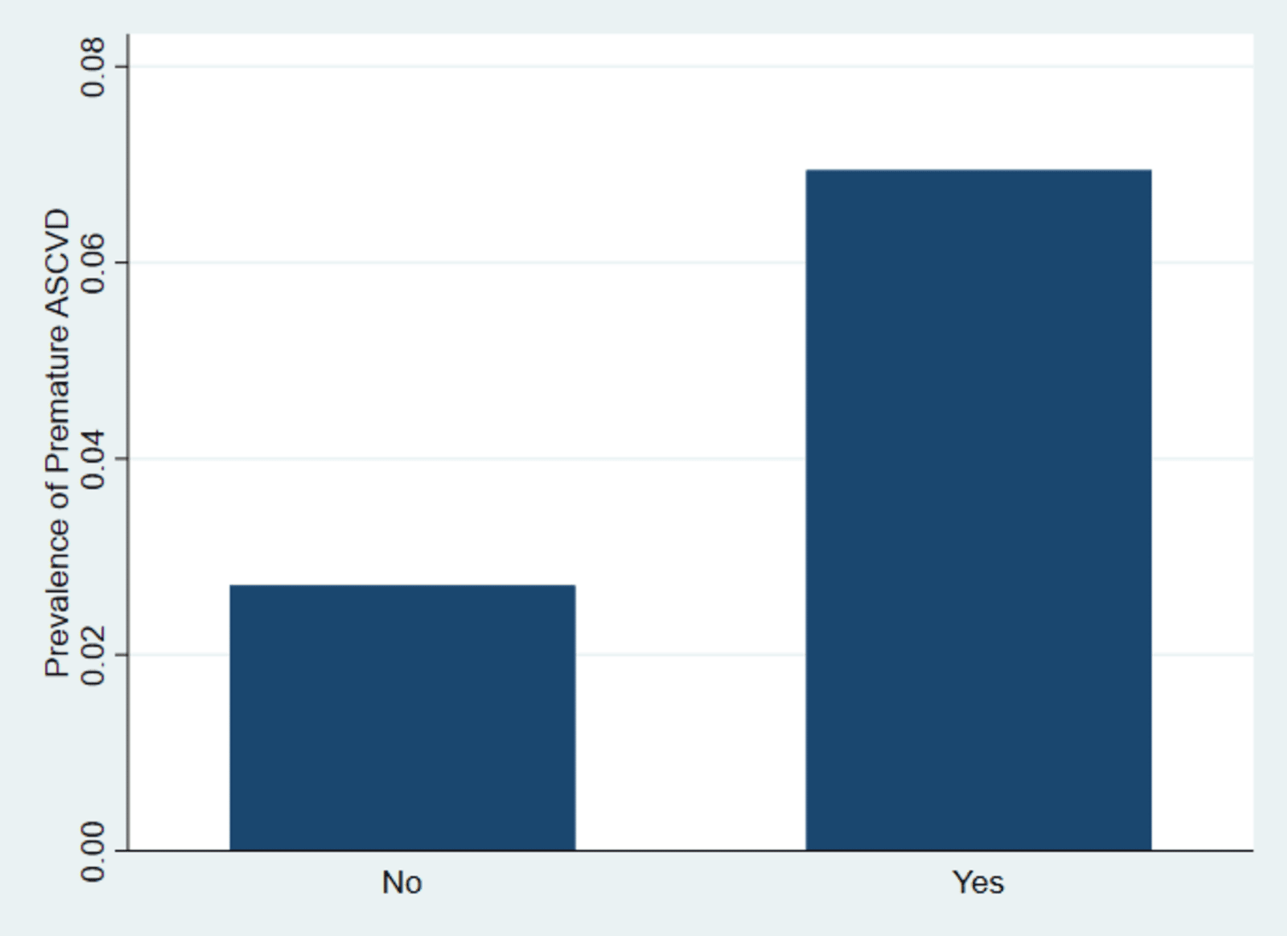
Prevalence of premature atherosclerotic cardiovascular disease (ASCVD) by albuminuria status

Figure 1 illustrates the weighted prevalence of premature ASCVD by albuminuria status.

The results indicate a higher prevalence of premature ASCVD among participants with albuminuria compared to those without albuminuria.

## Discussion

In this study, albuminuria was associated with higher odds of premature ASCVD among U.S. adults. Participants with albuminuria had a higher prevalence of premature ASCVD and a greater burden of cardiometabolic risk factors, including hypertension, diabetes, smoking, and CKD. Individuals with albuminuria were older and had lower income-to-poverty ratios, indicating a gradient across both clinical and socioeconomic factors. These findings are consistent with existing evidence that identifies albuminuria as an early marker of cardiovascular risk. Prior studies have shown that albuminuria reflects vascular injury and is associated with cardiovascular events even in the absence of traditional risk factors [7,9]. The observed association between albuminuria and premature ASCVD aligns with literature demonstrating that albuminuria predicts adverse cardiovascular outcomes across different populations [8,16]. The higher prevalence of risk factors among individuals with albuminuria is also in line with reports that early-onset CVD is strongly linked to modifiable risk profiles, including hypertension, diabetes, and smoking [2,3]. Differences across racial and ethnic groups, particularly the higher proportion of Non-Hispanic Black participants among those with albuminuria, are consistent with documented disparities in risk factor burden and access to preventive care [4,18].

Albuminuria serves as a clinically accessible biomarker that reflects underlying microvascular and endothelial dysfunction, processes that are pivotal in the pathogenesis of atherosclerosis and premature CVD. At the molecular level, endothelial cells form a selectively permeable barrier that regulates the passage of plasma components into the vessel wall. In states of endothelial dysfunction, this barrier integrity is compromised, resulting in increased vascular permeability. Albumin, normally restricted from traversing the endothelial barrier, leaks into the urine due to disruption of the glomerular filtration barrier and systemic microvascular injury [5, 6, 12].

This increased vascular permeability is mediated by alterations in endothelial tight junction proteins, cytoskeletal rearrangements, and oxidative stress-induced damage. Oxidative stress and inflammation, key contributors to endothelial injury, promote the expression of adhesion molecules and the release of pro-inflammatory cytokines, which amplify vascular dysfunction [13]. The presence of albuminuria thus signals a systemic endothelial phenotype characterized by impaired nitric oxide bioavailability, heightened reactive oxygen species, and dysregulated vascular homeostasis [11,12].

These molecular derangements facilitate the early development of atherosclerotic lesions by promoting lipid infiltration, leukocyte adhesion, and smooth muscle cell proliferation within the arterial intima. The resultant microvascular rarefaction and compromised perfusion further exacerbate tissue ischemia and organ dysfunction, accelerating disease progression [6,7,12]. Importantly, these processes occur even before the clinical manifestation of traditional cardiovascular risk factors, highlighting albuminuria as an early indicator of vascular pathology [10, 16].

In the context of premature ASCVD, the molecular interplay between endothelial dysfunction and albuminuria underscores a mechanistic pathway linking renal microvascular injury to systemic vascular disease. This connection elucidates why individuals with albuminuria exhibit higher odds of premature ASCVD, independent of established risk factors such as hypertension and diabetes [7,8,16]. Furthermore, the relationship between low-grade albuminuria and subclinical cardiac dysfunction, such as diastolic impairment, suggests that endothelial perturbations have direct consequences on myocardial structure and function, particularly in younger populations [14, 15, 17].

Current clinical guidelines in the US emphasize the importance of early identification and management of cardiovascular risk factors to prevent premature disease. Screening for kidney disease using both eGFR and albuminuria is recommended as part of cardiovascular risk assessment, particularly in individuals with hypertension or diabetes. Albuminuria is recognized as a marker of target organ damage and is incorporated into risk stratification frameworks for CVD. Evidence indicates that even low levels of albuminuria are associated with increased cardiovascular risk, supporting its role in early detection [11,15]. Guidelines also highlight aggressive control of blood pressure, glycemic status, and lifestyle factors such as smoking cessation to reduce cardiovascular risk. The findings of this study are consistent with these recommendations, as albuminuria was associated with a higher prevalence of established risk factors and with premature ASCVD.

Several biological mechanisms may explain the observed association between albuminuria and premature ASCVD. Albuminuria is considered a marker of systemic endothelial dysfunction, which contributes to the development of atherosclerosis [6,12]. Impairment of the endothelial barrier leads to increased vascular permeability and promotes inflammatory pathways that are central to plaque formation. In addition, albuminuria is associated with oxidative stress and chronic inflammation, both of which are linked to vascular damage and progression of CVD [13]. Studies have also shown that albuminuria is related to structural and functional cardiac changes, including left ventricular hypertrophy and diastolic dysfunction [14]. These pathways provide a plausible link between kidney injury and CVD, particularly in earlier stages where clinical disease may not yet be evident. The higher prevalence of CKD among individuals with albuminuria in this study further supports the interconnected relationship between renal and cardiovascular systems.

Strengths and limitations of the study

This study has several strengths. The use of NHANES data allowed for a large, nationally representative sample with standardized measurements and detailed information on clinical and sociodemographic factors. The analysis incorporated survey weights, strata, and clustering, which support generalizability to the U.S. adult population. Several limitations should be considered. First, the cross-sectional design precludes causal inference and limits the ability to establish temporal relationships. Second, the use of self-reported ASCVD outcomes may introduce recall and misclassification bias. Third, albuminuria was assessed using a single measurement, which may not fully capture variability over time. Fourth, residual confounding remains possible due to unavailable variables such as lipid levels, medication use, and lifestyle factors. Additionally, the use of complete case analysis may introduce selection bias if missingness is not completely random. These limitations should be considered when interpreting the findings. Future research should consider longitudinal designs to better assess temporal relationships and incorporate additional clinical measures to refine risk assessment.

## Conclusions

In this nationally representative cross-sectional study, albuminuria was associated with higher odds of premature ASCVD among U.S. adults. Individuals with albuminuria also demonstrated a greater burden of established cardiovascular risk factors. These findings support the role of albuminuria as a clinically relevant marker of cardiovascular risk and highlight its potential value in risk stratification, particularly among populations at risk for early CVD. The observed associations should be interpreted in the context of potential residual confounding and measurement limitations. From a public health perspective, these results support consideration of albuminuria in risk assessment frameworks. Further longitudinal studies are needed to better characterize these relationships and their implications for cardiovascular outcomes.
